# Melatonin blocks inhibitory effects of prolactin on photoperiodic induction of gain in body mass, testicular growth and feather regeneration in the migratory male redheaded bunting (*Emberiza bruniceps*)

**DOI:** 10.1186/1477-7827-2-79

**Published:** 2004-11-26

**Authors:** Amit K Trivedi, Sangeeta Rani, Vinod Kumar

**Affiliations:** 1 Department of Zoology, University of Lucknow, Lucknow 226 007, India

## Abstract

Little is known about how hormones interact in the photoperiodic induction of seasonal responses in birds. In this study, two experiments determined if the treatment with melatonin altered inhibitory effects of prolactin on photoperiodic induction of seasonal responses in the Palearctic-Indian migratory male redheaded bunting *Emberiza bruniceps*. Each experiment employed three groups (N = 6–7 each) of photosensitive birds that were held under 8 hours light: 16 hours darkness (8L:16D) since early March. In the experiment 1, beginning in mid June 2001, birds were exposed to natural day lengths (NDL) at 27 degree North (day length = ca.13.8 h, sunrise to sunset) for 23 days. In the experiment 2, beginning in early April 2002, birds were exposed to 14L:10D for 22 days. Beginning on day 4 of NDL or day 1 of 14L:10D, they received 10 (experiment 1) or 13 (experiment 2) daily injections of both melatonin and prolactin (group 1) or prolactin alone (group 2) at a dose of 20 microgram per bird per day in 200 microliter of vehicle. Controls (group 3) received similar volume of vehicle. Thereafter, birds were left uninjected for the next 10 (experiment 1) or 9 days (experiment 2). All injections except those of melatonin were made at the zeitgeber time 10 (ZT 0 = time of sunrise, experiment 1; time of lights on, experiment 2); melatonin was injected at ZT 9.5 and thus 0.5 h before prolactin. Observations were recorded on changes in body mass, testicular growth and feather regeneration.

Under NDL (experiment 1), testis growth in birds that received melatonin 0.5 h prior to prolactin (group 1) was significantly greater (P < 0.05, Student Newman-Keuls test) than in those birds that received prolactin alone (group 2) or vehicle (group 3). Although mean body mass of three groups were not significantly different at the end of the experiment, the regeneration of papillae was dramatically delayed in prolactin only treated group 2 birds. Similarly, under 14L:10D (experiment 2) testes of birds receiving melatonin plus prolactin (group 1) and vehicle (group 3) were significantly larger (P < 0.05, Student Newman-Keuls test) than those receiving prolactin alone (group 2). Also, birds of groups 1 and 3, but not of group 2, had significant (P < 0.05, 1-way repeated measures Analysis of Variance) gain in body mass. However, unlike in the experiment 1, the feather regeneration in birds of the three groups was not dramatically different; a relatively slower rate of papillae emergence was however noticed in group 2 birds. Considered together, these results show that a prior treatment with melatonin blocks prolactin-induced suppression of photoperiodic induction in the redheaded bunting, and suggest an indirect role of melatonin in the regulation of seasonal responses of birds.

## Background

In many birds, day length regulates seasonal changes in fattening and body mass gain, gonadal growth and development, molt, and plasma levels of several hormones, including luteinizing hormone (LH), prolactin and melatonin [1-4]. There occurs some degree of phase-relationship among various photoinduced events. For example, photoperiodically induced rise in LH coincides with the onset of breeding [1,2], and rise in prolactin coincides with the late breeding and early post-breeding periods [4,5]. During laying and incubation stages of the reproductive cycle plasma prolactin levels increase dramatically by 100 to 150 folds [6]. High prolactin levels in late breeding season are implicated in the development of reproductive photorefractoriness and postnuptial molt in birds [4,7]. Circulating melatonin levels also undergo seasonal changes. High melatonin levels in the summer months and low melatonin levels in the winter months coincide, respectively, with the breeding and non-breeding phases of the reproductive cycle in long day breeding birds [3]. Although not known in birds, Lincoln and Clarke [8] provide evidence that melatonin acts directly within the pituitary to regulate photoperiod-induced changes in prolactin secretion in seasonally breeding Soay sheep.

Previous studies on how hormones interact in photoperiodic induction of seasonal responses in birds have yielded conflicting results. A number of early findings show prolactin acting both as pro- and anti-gonadal in birds exposed to stimulatory day lengths [9-11]. However, in many birds high plasma prolactin levels are associated with decreased gonadal activity and LH levels [6,10-13]. In their recent review Blache and Sharp [4] conclude that prolactin is involved in the regulation of avian reproduction by providing inhibitory inputs to the hypothalamo-hypophyseal-gonadal axis.

The production and secretion of melatonin encodes a photoperiodic calendar to birds as they exhibit changes in both the duration and amplitude of melatonin secretion corresponding to the duration of night length/ day length [3,14,15]. Also, melatonin is part of the birds' multioscillatory circadian system and helps maintain a robust and stable phase relationship among different internal circadian oscillators, and photoperiodic induction of seasonal responses in birds is mediated by the circadian system [16-21]. Interestingly, however, a direct role of melatonin in avian photoperiodism is not explicitly found. Most studies negate the role of pineal/ melatonin in photoperiodic induction of seasonal responses in birds (for references see [17]). We propose that the role of melatonin in avian photoperiodism is indirect. Melatonin modulates sensitivity of the circadian response system to a stimulatory photoperiod, and/ or influences downstream the photoperiod-induced effects by interacting with other hormones released in response to stimulatory photoperiods [17,19]. We sought to investigate this by examining the effects of exogenous melatonin on prolactin-induced suppression of photoperiodic response in a migratory bird species, the redheaded bunting (*Emberiza bruniceps*), in which melatonin is not directly involved in the photoperiodic time measurement based on circadian rhythm of photosensitivity [22,23]. Previous studies show that prolactin administered subcutaneously at a dose of 100 μg day^-1 ^suppresses ovarian response in buntings subjected to long days [10]. In this study, we specifically determined if the administration of melatonin 0.5 h before prolactin blocks the prolactin-induced suppression of the photoperiodic induction of gain in body mass, testicular growth and development, and feather regeneration in the redheaded bunting exposed to stimulatory day lengths.

## Methods

**Figure 1:**
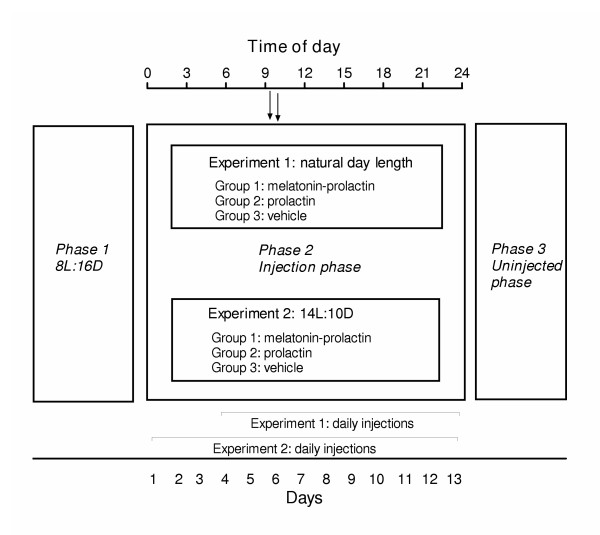
Experimental design. Both experiments had three phases: phase 1- pretreatment with 8 hours light: 16 hours darkness (8L:16D); phase 2- injection phase under natural day length (experiment 1) or 14L:10D; phase 3- uninjected phase. Arrows on top indicate time of injections (left: zeitgeber time (ZT) 9.5 – melatonin; right: ZT 10 – prolactin or vehicle; ZT 0 = the time of sunrise for the experiment 1, the time of onset of light for the experiment 2). Lines at the base of phase 2 indicate number of days of injection. The experiment 1 was terminated after 23 days and the experiment 2 was terminated after 22 days from the beginning of the phase 2.

We used adult male redheaded bunting caught in late February from the overwintering flock at 25°N. Buntings are migratory finch that breed in summer in west Asia and east Europe (~ 40°N) and overwinter in India. Birds were held outdoors and acclimatized to captive conditions for two weeks, and then brought indoors and maintained on short days (8 hours light: 16 hours darkness, 8L:16D) until subjected to experiments. Under short days, buntings do not fatten, and remain reproductively immature and responsive to photostimulation [24]; birds pretreated with short days are referred to as the photosensitive birds throughout this manuscript. Two experiments were performed as per experimental design detailed in the figure 1, and in accordance with the guidelines in the Principles of Animal Care.

### Experiment 1

This experiment began in mid June 2001. Photosensitive birds maintained indoors on 8L:16D since early March were brought outdoors in the aviary and exposed to natural day lengths (NDL) at 27°N (day length = ~ 13.8 h, sunrise to sunset). After three days of acclimatization, they were divided in three groups (N = 6 each). Beginning on day 4, they received subcutaneous injections once daily for 10 days as follows: group 1- first melatonin and 0.5 h later prolactin; group 2- prolactin alone; group 3- vehicle (control). After 10 consecutive injections (days 4–13), birds were left uninjected for the next 10 days (day 14–23). The experiment was terminated on day 24.

### Experiment 2

To confirm results of the experiment 1, we performed the experiment 2 under artificial conditions providing light-dark (LD) cycles corresponding to that was available outdoors (NDL) to birds of the experiment 1. This experiment began in the second week of April 2002. Three groups (N = 6–7) of photosensitive birds were subjected to 14L:10D. Beginning on day 1, they received 13 injections (days 1–13) as in the experiment 1: group 1- first melatonin and 0.5 h later prolactin; group 2- prolactin alone; group 3- vehicle (control). Thereafter, birds were left uninjected for the next 9 days (days 14–22). The experiment was terminated on day 23.

Melatonin was administered at zeitgeber time 9.5 (ZT 0 = the time of sunrise in the experiment 1; the time of light on in the experiment 2) in view of our previous study [23] and several other observations [17] showing that melatonin or vehicle given alone at this time of day does not affect photoperiodic induction of gain in body mass and testicular recrudescence in the redheaded bunting, although around this time of day melatonin administration affects photoperiodic induction in mammals [25]. The prolactin was administered at ZT 10; group 1 birds thus received prolactin 0.5 h after melatonin. Vehicle was administered to birds of group 3 at ZT 10. Thus, the timing of prolactin and vehicle injections was decided in relation to the timing of the melatonin injection that itself was timed in relation to the timing of sunrise or the timing of light on.

In both experiments, melatonin and prolactin were administered each at a dose of 20 μg bird^-1 ^day^-1 ^in 200 μl injection volume. Prolactin was obtained from Sigma Chemical Co. USA (Luteotropic hormone; L-6520, Lot 120K1606) and melatonin from Genzyme Fine Chemicals Ltd., Haverhill, Suffolk, UK). Melatonin injections were prepared as described by Kumar [26]. Briefly, a known amount of melatonin was dissolved in 100% ethanol and diluted in saline (0.9 % NaCl) such that each injection in 200 μl volume was 0.1 % ethanolic saline containing 20 μg of hormone. Prolactin was dissolved directly in saline yielding 20 μg per 200 μl of injection volume. Controls received 200 μl injection of 0.1% ethanolic saline (vehicle).

We measured the effects on changes in body mass, testis size and regeneration of feather papillae. Body mass and testis size were measured at the beginning (day 0, the day before injections began), in the middle (body mass only) and at the end of the experiments. Birds were weighed on a top pan balance providing accuracy nearest to 0.1 g. In view of the findings that the fattening accounts for the most of the gain in body weight in photostimulated passerine birds [27,28], in the present study we considered body mass of photostimulated redheaded bunting, a passeriform, reflecting the fat deposition. The dimensions of the left testis were recorded when birds were laparotomised under local anesthesia (for details see [29]), and testis volume was calculated from 4/3π*ab*^2^, where *a *and *b *denote half of the long and short axes, respectively. Feather papillae regeneration was recorded as follows. On day 1 of the experiment, feathers in a specific area on the left chest were plucked. A permanent ink marker marked an area on bare epidermis measuring 1 cm^2^. Beginning 24 h after the first injection, the number of papillae emerged from the epidermis were counted daily throughout the experiment 1 or till 4 days after the last injection in the experiment 2, and scored subjectively as outlined by Boswell [30]. Briefly the scoring was done as follows: 0- missing feather, 1- a papilla emerging, 2- a papilla grown up to one-third of full size, 3- a papilla grown up to two-third of full size, 4- a papilla grown more than two-third of full size but still not complete, and 5- a completely grown feather papilla. The feather papillae scores were limited to first 50 feathers emerged within the marked area, and so total papillae score for an individual bird ranged from 0–250.

Food and water were available *ad libitum*. In an artificial LD cycle, light was provided by white compact fluorescent lamps at ~ 500 lux. Data are presented as mean ± SE. They were analyzed using one-way analysis of variance (1-way ANOVA) with or without repeated measures, followed by post-hoc tests if ANOVA indicated a significance of difference. 1-way repeated measure ANOVA was used to compare data generated from the same group as a function of time, and 1-way ANOVA was used to compare data of different groups at one observation. Two-way (2-way) ANOVA was used to analyze data when two factors were considered together, for example the effect of the treatment and duration of the treatment. Significance was taken at *P *< 0.05. In the experiment 1, one bird of group 1 and two birds of group 2 died, and their data are excluded from the statistical analyses.

## Results

### Experiment 1

**Figure 2:**
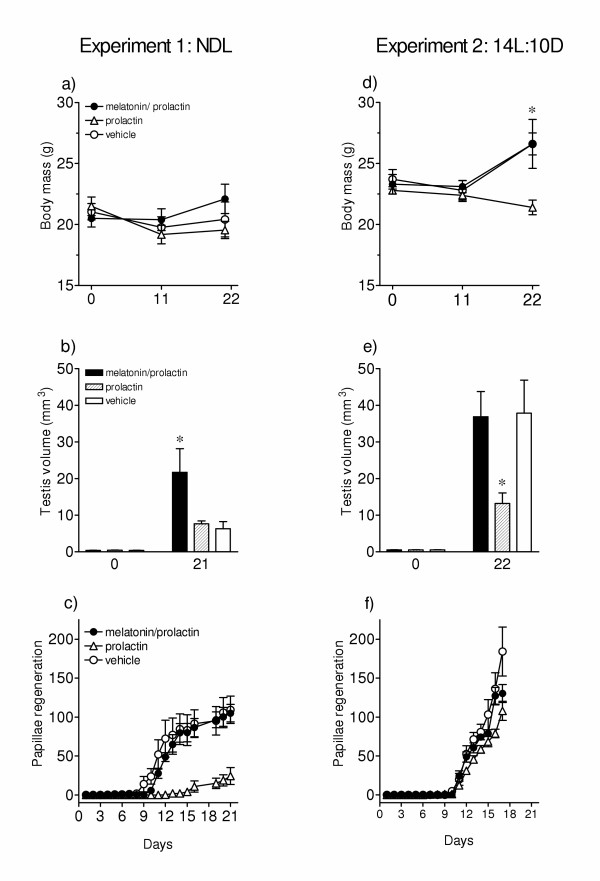
Mean (± SE) body mass, testis volume and feather papillae regeneration in response to treatment with melatonin and prolactin (group 1), prolactin alone (group 2), or vehicle (group 3) in the redheaded bunting (*Emberiza bruniceps*) subjected to natural day lengths at 27°N (experiment 1: a-c) for 23 days (from mid-June to early July) or to artificial day length (14L:10D; experiment 2: d-f) for 22 days. Birds were injected with exogenous hormones at a dose of 20 μg bird^-1 ^day^-1 ^in 200 μl of vehicle daily for first 10 days in the experiment 1 and for first 13 days in the experiment 2, and thereafter they were left uninjected. Controls received similar volume of vehicle. All injections except melatonin were made at the zeitgeber time 10 (ZT 0 = the time of sunrise for the experiment 1; the time of onset of light for the experiment 2); melatonin was injected at ZT 9.5. Day 0 on X-axis refers to the day before first injection. Under NDL, one bird of group 1 and two birds of group 2 died, and their data are excluded. Asterisk indicates the significance of difference at *P *< 0.05.

Results are shown in figure 2a,2b,2c. There was no significant change in body mass in birds of all the three groups during the treatment period (Fig. 2a). Testes were however stimulated in all birds but the mean testis volume at the end of the experiment was different among the three groups (*F*_(2,12) _= 4.656, *P *= 0.0319; 1-way ANOVA). Testes were significantly larger (*P *< 0.05, Newman-Keuls test) in birds that received melatonin prior to prolactin (group 1) compared to those that received prolactin alone (group 2) or vehicle (group 3) (Fig. 2b). The rate of regeneration of feathers was not significantly different between birds of groups 1 and 3 (*F*_(1,180) _= 3.3.19, P = 0.0701; 2-way ANOVA) although in group 1 birds the emergence of the first papilla was delayed by at least a day (Fig. 2c). Whereas in the group 1 the first papilla in a bird was found on day 5 and in all birds by day 11, in the group 3 the first papilla in a bird was found on day 4 and in all birds by day 10. Also, mean papillae scores during first some days were relatively smaller in group 1 compared to group 3. In group 2 birds that received prolactin alone the first papilla emergence in a bird was found on day 12, and hence papillae regeneration was dramatically delayed as compared to birds in group 1 (*F*_(17,144) _= 15.26, P < 0.0001; 2-way ANOVA) and group 3 (*F*_(17,144) _= 16.49, P < 0.0001; 2-way ANOVA) (cf. Fig. 2c).

### Experiment 2

Figure 2d,2e,2f show results from the experiment 2. There was a significant gain in body mass in birds of groups 1 and 3 which received melatonin plus prolactin and vehicle, respectively (group 1: *F*_(2,10) _= 12.16, *P *= 0.0021; group 3: *F*_(2,12) _= 6.978, *P *= 0.0098; 1-way RM ANOVA; Fig. 2d), but not in birds of group 2 which received prolactin alone (*F*_(2,12) _= 0.2839, *P *= 0.7379; 1-way RM ANOVA; Fig. 2d). Hence at the end of the experiment, there was a significant difference in the response of body mass among the three groups (*F*_(2,17) _= 3.857, *P *= 0.0416; 1-way ANOVA). Although testes were stimulated in all groups (Fig. 2e), the size attained at the end of the experiment was different among different groups (*F*_(2,17) _= 4.343, *P *= 0.0299; 1-way ANOVA). Testes grew to full size in birds of groups 1 and 3, and hence were significantly larger (*P *< 0.05, Newman-Keuls test) than those of group 2 in which they grew to less than half-maximal size (Fig. 2e). Unlike the experiment 1, the feather regeneration was not dramatically different among the three groups (Fig. 2f). Nonetheless, the rate of papillae regeneration in birds of group 2 that received prolactin alone was slower as compared to group 1 (*F*_(14,150) _= 3.9550, P < 0.0485; 2-way ANOVA) and group 3 (*F*_(14,165) _= 24.76, P < 0.0001; 2-way ANOVA) (cf. Fig. 2f). By day 17, however, all individuals regardless of the treatment had shown papillae emergence (Fig. 2f).

## Discussion

The present results confirm a previous finding on female redheaded buntings [10] that exogenous prolactin suppresses the photoperiodic induction of body mass gain and testis recrudescence under long days. In general, the effects of prolactin on body mass and testes found in the present study are consistent with the evidence that high prolactin levels during late breeding phase decrease fat stores [31] by affecting lipid metabolism via increasing lipoprotein lipase activity in the adipocytes [32] and induce testicular regression by inhibiting the hypothalamo-hypophyseal-gonadal axis [4,7,13]. Prolactin-induced suppression of papillae emergence in buntings is also consistent with the suggested role of high prolactin inducing defeathering and postnuptial molt [4,7]. High prolactin levels induced by prolactin administration thus seem producing a physiological condition comparable to late phase of the gonadal cycle, both in terms of declining body mass, reduced testicular activity and defeathering indicated by suppression of the emergence of feather papillae (Fig. 2).

Of more interest is however that a prior treatment with melatonin blocks prolactin-induced suppression of photoperiodic induction in the redheaded bunting (Fig. 2). Birds administered with melatonin 0.5 h prior to prolactin showed photoperiodic induction similar to that of controls (experiment 2; Fig. 2d,2e,2f). Data on feather regeneration also support this. Melatonin administration blocked the suppression of papillae emergence by exogenous prolactin (cf. Fig. 2e). It is not understood however from these experiments how melatonin acts to restore photoperiodic response in prolactin-treated birds, but we can offer some plausible explanations. One is that melatonin reduces circulating prolactin levels either directly by acting on pituitary prolactin producing cells, as reported in fish [33,34], or indirectly by affecting the release of hypothalamic dopamine (DA) and vasoactive intestinal peptide (VIP) [35,36]. There is increasing evidence from both *in vivo *and *in vitro *experiments that hypothalamic VIP acts as prolactin releasing factor [35] and its secretion is photoperiodically regulated in birds [35,36]. Both the DAergic and VIPergic systems interact in regulation of prolactin secretion in birds [35]. It is to be investigated if there is a relationship between the melatonin and VIP, but from studies on avian retinal system there is evidence for an inverse relationship between the melatonin and dopamine (DA) [37]. Additionally, melatonin acts directly within the pituitary to regulate prolactin secretion in seasonally breeding photoperiodic Soay sheep [8], and melatonin stimulates dopamine release from tuberoinfundibular dopaminergic neurons resulting into the suppression of serum prolactin levels in rats [38]. Current results (Fig. 2) that when photostimulated buntings receiving both melatonin and prolactin had greater body mass and larger testes than those receiving prolactin are consistent with one or the other of the above explanations.

A second possibility is that exogenous melatonin changes phase-relationship between daily rhythms of endogenous endocrine rhythms, and this somehow enhances sensitivity of the circadian response system to stimulatory effects of long day lengths. Testicular response in birds of the experiment 1 supports this (Fig. 2b). Birds that received both melatonin and prolactin (group 1) had significantly larger testes (*P *< 0.05, Student Newman-Keuls test) than those received prolactin alone (group 2) or vehicle (group 3). A role of melatonin in enhancing responsiveness of the photoperiodic response system is shown in an experiment on the blackheaded bunting (*Emberiza melanocephala*), an allied species that shares breeding and wintering grounds with the redheaded bunting. In blackheaded buntings exposed to 11.75L:11:25D of red light (650 nm), testes grew significantly larger in individuals that carried implants filled with melatonin compared with those that carried empty implants [39].

However, one observation of the present study is not entirely consistent. Controls of the experiment 1 had significantly (*P *< 0.05, Student t-test) smaller testes than those of the experiment 2. This occurred perhaps because of one or both of the following reasons. First, there was a difference in the lighting environment between two experiments, both in the shape of LD cycle (saw-tooth shape in NDL versus square-wave shape in 14L:10D) and intensity of light period (gradually changing intensity for ~ 13.8 h daylight outdoors underneath opaque roof of the aviary in NDL (light intensity in the aviary during the experiment ranged from 92.3 ± 13.4 lux at sunrise to several thousand lux during day to 56.2 ± 5.9 lux at the time of sunset) versus a continuous ~ 500 lux intensity for 14 h within the photoperiodic chambers in 14L:10D). It is reported that the duration of photoperiod and light intensity do affect photoperiodic induction of body mass and testis recrudescence in the blackheaded bunting [24]. Second, high temperatures (> 40°C) outdoors during mid June – early July may have caused rise in endogenous prolactin levels [40], similar to those in group 2 that received exogenous prolactin, and this may have suppressed the photoperiodic induction (cf. Fig 2a,2b).

The present study indicates that melatonin could be involved indirectly in regulation of photoperiod-induced seasonal responses in the redheaded bunting by modulating effects of other hormones such as the prolactin. This appears consistent with another finding on this species [23] suggesting effects of melatonin on temporal phasing of the testicular cycle (individuals that carried implant filled with melatonin peaked in testicular growth one-month later compared to those that carried empty implant) and not on the initiation of testicular recrudescence. However, unlike in several birds (for references see [17]) in which melatonin fails to produce an effect on testicular growth, a few reports do exist in the literature showing direct effects of melatonin administration on gonadal activity. In migratory European quail (*Coturnix coturnix*), for example, melatonin given in drinking water influences the reproductive cycle [41]. Daily melatonin injections inhibited testicular recrudescence in lal munia (*Estrilda amandava*) [42], and caused significant involution of enlarged testes of breeding season in both the blossomheaded parakeet (*Psittacula cyanocephala*) and the Indian weaver bird (*Ploceus philippinus*) [43]. Inhibitory effects of pineal on hypothalamo-hypophyseal-gonadal axis is reported in the Indian weaver bird (*Ploceus philippinus*) [44] although a recent study in which reproductively active individuals were implanted with melatonin did not support the antigonadal effect of melatonin [23]. Differences in the effects of melatonin probably reflect diversity of the avian photoperiodic system. It will be interesting therefore to further examine species showing divergent effects of melatonin to unravel the diversity of the role of melatonin in photoperiod-induced seasonal responses in birds.

## Conclusion

The present results show that a prior treatment with melatonin blocks prolactin-induced suppression of photoperiodic induction in the migratory redheaded bunting. How melatonin acts to negate the effects of prolactin is unclear. Whatever is the actual mechanism of action, the current result provide evidence that melatonin modulates photoperiodic induction of seasonal responses in birds by interacting with prolactin. Whether such effect will vary during different seasons of the year remains to be investigated.

## Authors' contributions

AKT and SR carried out the experiments and prepared the first draft of the manuscript. VK supervised the experiments and the final version of the manuscript. The study was conceived by VK but then the experiments were discussed jointly. All the authors approved the final manuscript.
